# H_2_O Derivatives Mediate CO Activation in Fischer–Tropsch Synthesis: A Review

**DOI:** 10.3390/molecules28145521

**Published:** 2023-07-19

**Authors:** Shuai Zhang, Kangzhou Wang, Fugui He, Xinhua Gao, Subing Fan, Qingxiang Ma, Tiansheng Zhao, Jianli Zhang

**Affiliations:** 1 State Key Laboratory of High-Efficiency Utilization of Coal and Green Chemical Engineering, College of Chemistry & Chemical Engineering, Ningxia University, Yinchuan 750021, China; 2 School of Materials and New Energy, Ningxia University, Yinchuan 750021, China

**Keywords:** Fischer–Tropsch synthesis, CO activation, H_2_O molecule-assisted, H-assisted, OH-assisted, O-assisted

## Abstract

The process of Fischer–Tropsch synthesis is commonly described as a series of reactions in which CO and H_2_ are dissociated and adsorbed on the metals and then rearranged to produce hydrocarbons and H_2_O. However, CO dissociation adsorption is regarded as the initial stage of Fischer–Tropsch synthesis and an essential factor in the control of catalytic activity. Several pathways have been proposed to activate CO, namely direct CO dissociation, activation hydrogenation, and activation by insertion into growing chains. In addition, H_2_O is considered an important by-product of Fischer–Tropsch synthesis reactions and has been shown to play a key role in regulating the distribution of Fischer–Tropsch synthesis products. The presence of H_2_O may influence the reaction rate, the product distribution, and the deactivation rate. Focus on H_2_O molecules and H_2_O-derivatives (H*, OH* and O*) can assist CO activation hydrogenation on Fe- and Co-based catalysts. In this work, the intermediates (C*, O*, HCO*, COH*, COH*, CH*, etc.) and reaction pathways were analyzed, and the H_2_O and H_2_O derivatives (H*, OH* and O*) on Fe- and Co-based catalysts and their role in the Fischer–Tropsch synthesis reaction process were reviewed.

## 1. Introduction

Fischer–Tropsch synthesis (FTS) is a catalytic chemical reaction in which syngas, a mixture of carbon monoxide (CO) and hydrogen (H_2_) derived from coal, biomass, or natural gas, is converted into mainly hydrocarbons (light olefins, liquid fuels, linear α-olefins, etc.), as well as oxygenated compounds (alcohols, aldehydes, ketones, and acids) [1,2,3,4]. The synthesis of methanation, paraffins, olefins, and oxygenated compounds can be represented by Equations (1)–(4). Compared with other metals, Fe- and Co-based catalysts are suitable and often used for FTS due to their high activity and target products selectivity, low water gas shift (WGS) activity, and relatively low cost [5,6,7]. At the same time, Fe- and Co-based catalysts have differences such as the product distribution being very dependent on the type of catalysts (Fe, Co) used and on the reaction conditions. For instance, Fe has a high water gas shift activity and is used when the syngas is produced from coal, i.e., when the water gas shift reaction is desirable due to low H_2_/CO ratios in this syngas; Co is the preferred catalyst for the FTS of long chain paraffins from natural gas due to their high activity and selectivity, low water gas shift activity, and comparatively low price. To explain the production and distributions of different products, many researchers have worked to identify and clarify the mechanism underlying the FTS response. However, the details of some mechanisms remain unclear and speculative [8,9].

Methanation: CO + 3H_2_ → CH_4_ + H_2_O (1)

Olefins: 2nH_2_ + nCO → C_n_H_2n_+ nH_2_O (2)

Paraffins: (2n+1)H_2_ + nCO → C_n_H_2n+2_ + nH_2_O (3)

Oxygenates: 2nH_2_ + nCO → C_n_H_2n+2_O + (n − 1)H_2_O (4)

**Figure 1:**
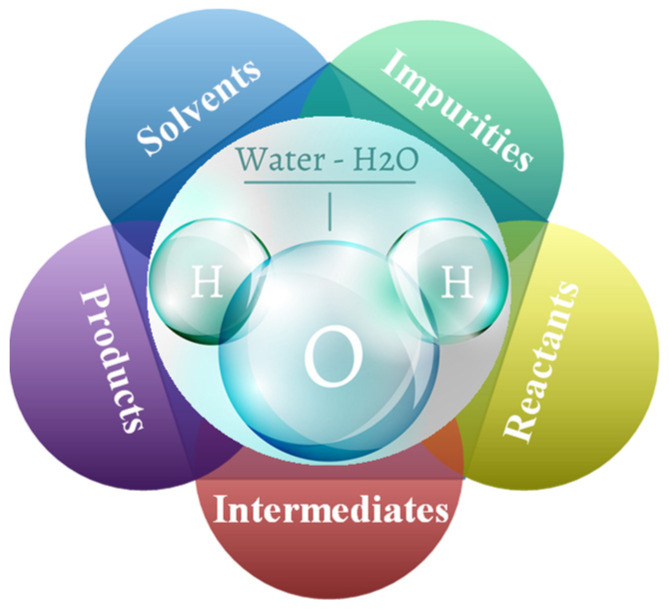
The role of water in the Fischer–Tropsch synthesis reaction.

In a series of recent papers on FTS deactivation [10], selectivity towards higher hydrocarbons [11], and the influence of the catalyst supports [12], the H_2_O was found to be not a silent spectator but to play a significant role in all parts of FTS [1]. H_2_O can exist in all three physical states of matter, but in chemistry, on the surface of catalysts, the states of H_2_O are diverse and dynamic, depending on the surface types and the thermodynamic conditions. H_2_O is not only prevalent in most chemical reactions as solvents, impurities, reactants, intermediates, or products, but it also plays a special role in its promotion or inhibition as shown in Figure 1 [13,14,15,16,17,18,19,20,21]. The effect of H_2_O on catalyst performance was initially described by Minderhoud et al. [22] and Kim et al. [23]. The adsorption of H_2_O can create favorable conditions for a number of surface reactions. Several studies have been conducted to determine the mechanism of the H_2_O action and some viable theories have been proposed. For example, H_2_O can inhibit the secondary hydrogenation of primary olefins [24,25], oxidize exposed CO molecules during H_2_O treatment [26], alter the active carbon concentration [27], facilitate the transport of syngas and hydrocarbons within the particles [24], and the addition of reactive H_2_O and produced H_2_O during synthesis leads to higher selectivity for higher hydrocarbons [11,12,28,29,30,31,32]. Studies have demonstrated that H_2_O is always present in various forms during the FTS reaction, altering the activity and selectivity of the FTS in various ways [33,34,35,36].

It is well known that one of the undesirable byproducts of the FTS process is H_2_O, and several experimental studies have shown that both H_2_O generated during the reaction and H_2_O introduced from outside can reduce or increase the reaction activity [8,13,26,37]. For example, it was observed that H_2_O reversibly enhances the activity of some catalysts but not all. Reversible selectivity changes induced by H_2_O include decreased methane selectivity, higher product olefinicity at a given carbon number, and an increased fraction of higher molecular weight products [38]. The role of H_2_O in FTS reactivity, reaction pathway, and product distribution has been reported in the literature, but the effects of H_2_O and H_2_O derivation (H*, OH* and O*) on CO activation hydrogenation on Fe- and Co-based catalysts have hardly been reported in detail. Here, we only study the effect of H_2_O on CO dissociation activation on Fe- and Co-based catalysts. Elucidating the mechanism of CO activation on the surface of Fe- and Co-based catalysts is essential for understanding the initial process of FTS reaction. In this study, a comprehensive review of research activities on H_2_O derivatives mediating CO activation hydrogenation in FTS is presented. A detailed summary and analysis of the H_2_O molecule, H-assisted, OH-assisted, and O-assisted CO activation are presented, which helps to elucidate the effect of H_2_O. Finally, the field is presented for conclusions and outlook.

## 2. H_2_O Derivatives Mediate CO Activation

### 2.1. CO Dissociation Activation

Here, we present a short review on the mechanism of CO dissociation, which is regarded as the initial step in the FTS. As a very important and useful basic chemical, CO has found a wide range of applications in the energy society as well as in the production of value-added bulk and fine chemicals. To provide further insight into these key processes, it is essential to study the mechanisms of CO adsorption and dissociation on the surfaces of heterogeneous catalysts. More specifically, the adsorption and desorption of CO on Fe- and Co-based catalyst surfaces are very essential steps related to catalytic activity.

In order to fundamentally understand the process of FTS reaction, it is essential to investigate the mechanism of CO activation hydrogenation on the surface of Fe- and Co-based catalysts, and several pathways for CO activation have been proposed, including direct CO dissociation, hydrogenation, and insertion into the growth chain for activation [33,39,40,41,42,43,44,45]. It is generally accepted that CO dissociation is the rate-determining step [42,46]. Up to now, the general consensus derived from experimental and theoretical studies about the elementary steps involved in the FTS can be categorized into (a) initiation, which involves the CO dissociation and CH_x_ formation; (b) propagation via the C-C coupling reactions; and finally, (c) the termination and desorption of the hydrocarbons. This process involves contrary reactions of bond breaking and forming on the metal surfaces [39]. Although the mentioned mechanisms are detailed, the exact paths that explain the formation of transition species between reagents and products are not entirely understood. After reviewing the literature, we found out that for Fe- and Co-based catalysts surfaces, the direct dissociation and the H-assisted CO dissociation are the main proposed mechanisms [45]. Next, we will focus on the CO-activated dissociation part of the direct dissociation and H-assisted mechanisms.

**Figure 2:**
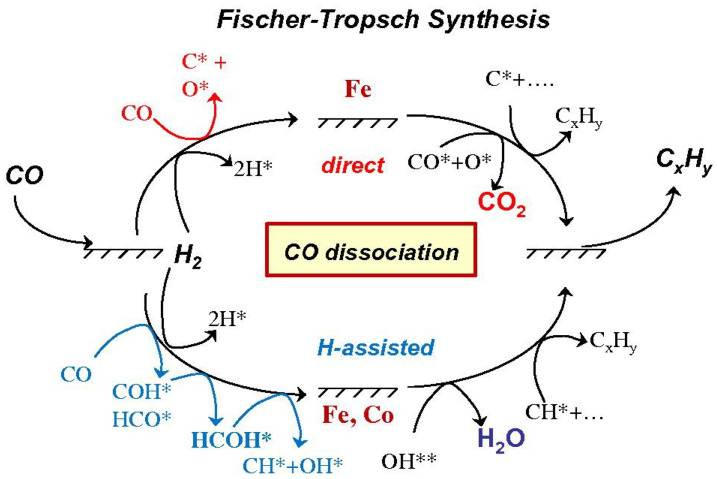
CO activation reacts with chemisorbed hydrogen before and after C-O bond cleavage on Fe and Co-based catalysts. Reproduced with permission from ref. [45]. Copyright (2010) Elsevier.

In general, the FTS can be described as a polymerization reaction, where three essential steps are identified: the first step is the initiation, which involves the adsorption of CO and H_2_ on the metal surface; the second step is the polymerization and chain formation; and the third step is products desorption [39]. As shown in Figure 2, direct dissociation pathways and H-assisted pathways have been proven to play an important role in kinetic-related CO dissociation steps on Fe- and Co-based catalysts. In the direct dissociation pathway, CO directly dissociate on the Fe surface to C* and O* species for subsequent reactions. In the H-assisted pathway, CO molecules are not directly dissociated on the surface of Fe- and Co-based catalysts, and CO forms COH* or HCO* intermediates with H* species. Intermediate species formed in these pathways can be continuous reactions, such as chemically adsorbing hydrogen (H*) on the surface of Fe- and Co-based surfaces to interact with CO* hydrogenated to form H_x_CO species, or directly forming OH* precursors, resulting in the preferential repulsion of H_2_O by O atoms in CO. On the Fe-based surface, CO* interacts with O* to form CO_2_. These assisted pathways represent the exclusive CO activation routes on Co surfaces and the predominant one on Fe catalysts at relevant FTS conditions. H-assisted pathways occur concurrently with unassisted CO dissociation on Fe-based catalysts with oxygen rejection as CO_2_ [45]. Both pathways produce intermediates with significant chain growth under the desired reaction conditions.

### 2.2. H_2_O Molecules-Assisted CO Activation

In recent years, there has been a growing interest in understanding reactions at the molecular level to improve energy efficiency, product selectivity and renewability of chemical processes, thus making the description of each reaction step a key aspect for improving such processes [4,8,10]. Adsorption and reaction of H_2_O on solid surfaces are fundamental ingredients for numerous chemical reactions in many practical applications such as heterogeneous catalysis, electrochemistry, and corrosion, to which various analytical techniques have been applied [36]. For most cases of heterogeneous catalysis, H_2_O is found to have an important role as a promoter or inhibitor, which is typically unrecognized or underestimated but is critical for understanding the essence of catalytic processes [21]. Particularly, FTS is often viewed as a sequence of reactions in which CO and H_2_ are dissociatively adsorbed and subsequently rearranged to form hydrocarbons and H_2_O [47]. The H_2_O molecules are thought to be a byproduct of the FTS reaction, but the presence of H_2_O has been shown to play an important role in regulating the distribution of products in FTS. We will analyze how H_2_O molecules affect CO dissociation activation. The mechanism responsible for the positive effects of H_2_O has been reported to facilitate the mass transfer of syngas and hydrocarbons to influence the kinetics of the reaction [25,48,49,50,51,52,53]. H_2_O molecules are adsorbed on contact with the surface of metallic Fe- and Co-based catalysts, and they can dissociate into OH* and H* species, as well as OH* radical into O* and H* species as intermediates in a subsequent reaction [8,54,55]. The role of H_2_O in FTS, such as the degree of adsorption and dissociation, chain initiation, chain growth, methanation, and olefins hydrogenation, are all possible effects of H_2_O molecules on the catalyst that may affect syngas conversion, hydrocarbon selectivity, FTS product distribution, and catalyst stability [8,39,54,56,57,58]. When H_2_O molecules dissociate and adsorb, they can polymerize and hydrogenate with CH_2_, leading to alkane chain growth [7,59]. H_2_O molecules inhibit the secondary hydrogenation of olefin products, which may be the result of competitive adsorption of H_2_O molecules [24,25].

**Figure 3:**
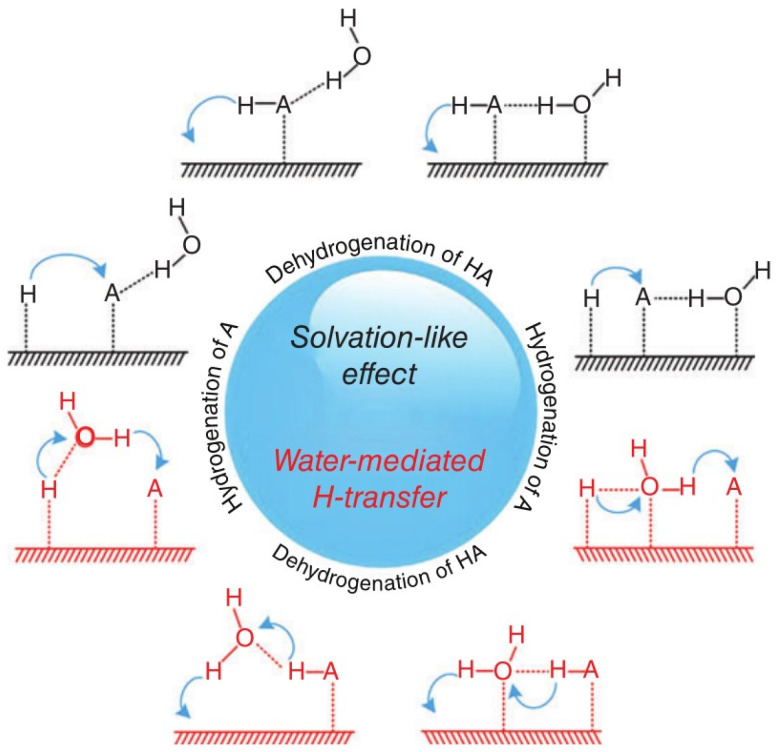
Schematic of the promotional role of water molecule: solvation-like effect (in black) and water-mediated H-transfer (in red). A refers to reactants of hydrogenation reactions, and H-A refers to reactants of dehydrogenation reactions. Reproduced with permission from ref. [21]. Copyright (2016) Wires.

**Figure 4:**
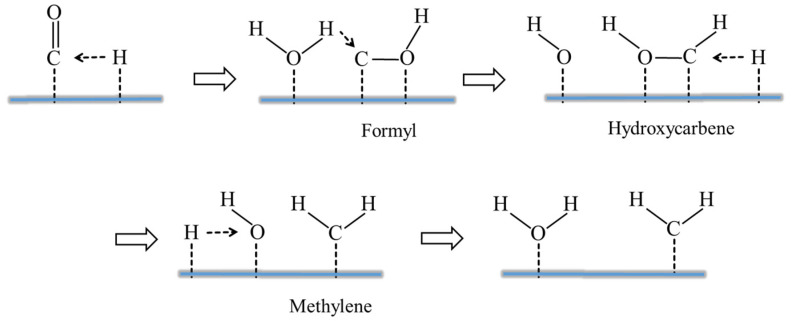
H_2_O- and H-assisted activation of CO to methylene. Reproduced with permission from ref. [65]. Copyright (2018) Springer Link.

This work only discusses the effect of H_2_O on the CO-activated hydrogenation process. The present results show that H_2_O has a dramatic effect on the dynamic equilibrium around C* species: the H_2_O increases the coverage of C* species by preferentially increasing the rate of CO activation, which also slightly affects the production of branched chain hydrocarbons and internal olefin isomers [60]. As shown in Figure 3, H_2_O may influence formate route: (1) as a H-source (H_2_O* reacts with HCO* to form *HCOH* and OH*); (2) as a “solvent” to stabilize the FTS for H-addition to the O atom of HCO* (through H-bonding to the primary O-H bonds); or (3) as a H-closing agent (as a H_2_O molecule or extended phase) for H* transfer to the O-atom in HCO* [21,61]. The HCO* intermediate corresponds to a lower barrier than the direct CO dissociation, since the formation of HCO* species is an endothermic process. As shown in Figure 4, the increase in CO activity in the presence of H_2_O may be explained by a direct interaction between the weak hydrogen bond of H_2_O and the oxygen of CO, reducing the barrier to dissociation of the CO barrier [27] and enhancing the surface coverage of polymeric intermediates [62]. Both direct adsorbate–adsorbate (e.g., weak hydrogen bonding of H_2_O to the oxygen of CO) and metal-mediated mechanisms (e.g., increased back-bonding to the π* orbital of CO) are explained by the interaction between CO and H_2_O [34,36,63,64]. Kim [23] found higher CO conversion and higher C_5_^+^ and lower methane selectivities when a small amount of external H_2_O was added during FTS on Co-based catalysts. Therefore, H_2_O molecules not only affect CO activation and dissociation in FTS reactions but also affect the reaction path and product distribution. This article reviews the intrinsic mechanism of the role of H_2_O in the surface reaction of Fe- and Co-based catalysts, so that researchers can better understand the H_2_O effect.

### 2.3. H-Assisted CO Activation

There are two parallel CO activation pathways on the surface of Fe and Co-based catalysts, which are unassisted activation and H-assisted activation [39,40,41,42,43,44,66]. Reaction paths and products vary under different reaction conditions. Here, we have reviewed experimental and theoretical evidence that the H-assisted pathway plays an important role in the kinetically relevant CO activation steps on both Fe- and Co-based catalysts under the required reaction conditions [67,68]. It is necessary to understand all possible competing and successive surface reactions starting from the H-assisted CO dissociation activation. In the FTS reaction, one H is derived from H_2_ in the syngas, and the other H from H_2_O produced [65]. This review is an attempt to understand the role of H-assisted CO activation and the initial stages of the formation of essential surface species such as CH*, CH_2_*, and OH* intermediates in FTS. Therefore, we summarize some key points that illustrate the importance of the H-assisted pathways for significant chain growth in the relevant CO activation steps over Fe- and Co-based catalysts under reaction conditions [45]. The results indicate that co-adsorption H* has a stabilizing effect in the system, while the increase in CO adsorption energy favors its reorganization.

**Figure 5:**
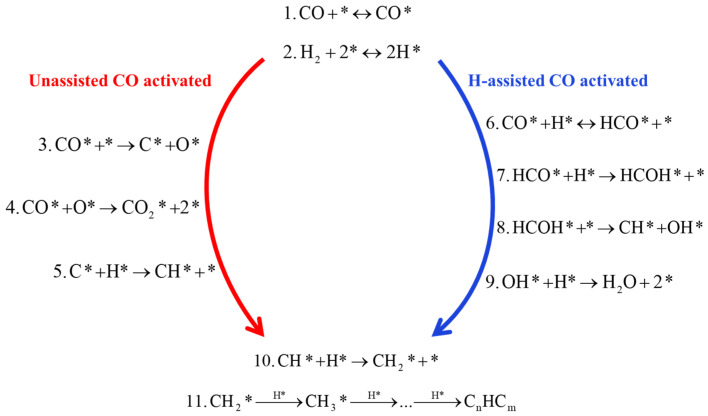
The H-assisted CO-activated hydrogenation in the FTS reaction elementary steps. Reproduced with permission from ref. [45]. Copyright (2010) Elsevier.

It has been previously reported that the preferential adsorption site for CO activation reaction occurs at hollow sites on the surface of Fe- and Co-based catalysts, forming CO* species [43,44] (step 1, Figure 5). The dissociative adsorption of H_2_ molecules requires the formation of H* species at two hollow adsorption sites on the Fe-based surface [69] (step 2, Figure 5). In the unassisted CO dissociation pathway, CO* is directly dissociated to form C* and O* species (step 3, Figure 5), and both C* and O* species are hydrogenated to form the intermediate products CH_2_ (carbene) and H_2_O, i.e., the carbide mechanism (step 4, Figure 5) [45]. The O* species formed in step 3 are removed via reaction with CO* as CO_2_ molecule (step 5, Figure 5). For H-assisted CO activation, Storch et al. [4,43] proposed a mechanism by which a hydrogen atom is added directly to the adsorbed CO* species, leading to the formation of formyl (HCO*) species (step 6, Figure 5). HCO* species formation or dissociation into CH* and O* was performed with the presence of an H* and a coadsorbed CO or an HCO* species and an empty neighbor site, respectively. Subsequently, the HCO* species undergoes hydrogenation on the O-atom to form hydroxycarbene (HCOH*) intermediates (step 7, Figure 5). The HCOH* intermediate is unstable and subsequently dissociates to form the chain growth monomer CH* and initiator OH* [39] (step 8, Figure 5). The OH* species formed in step 8 continue to hydrogenate and are scavenged as H_2_O molecules (step 9, Figure 5). This indicates that step 9 is the primary mechanism for the removal of OH* in the FTS reaction. Alternatively, OH* may react with CO* to form carboxyl (COOH*) species, which subsequently decompose to produce CO_2_ and H* [68]. DFT calculation indicates that the formation of COOH* has a higher activation energy barrier than the formation of H_2_O [68]. The unassisted CO activation and H-assisted activation pathways produce CH* species, and both pathways form CH* monomers that are successively hydrogenated to form CH_2_ and CH_3_ for alkyl chain growth (step 10 and 11, Figure 5). In the unassisted CO activation pathway, CO removes the chemisorbed oxygen (O*) into CO_2_ molecules, while the H-assisted CO activation pathway forms only H_2_O molecules. The results indicate that the presence of co-adsorbed hydrogen has a stabilizing effect in the system, increasing the adsorption energy of CO and facilitating its recombination. In these cases, the H-assisted route provides energetically more favorable alternatives [45,59]. Indeed, the dissociation of H_2_O molecules has the potential to various chemicals with other species on the surfaces.

### 2.4. OH-Assisted CO Activation

On the surface of metal oxide Fe- and Co-based catalysts, H_2_O could adsorb at both metal cations and oxygen anions or dissociate into two hydroxyl groups, one from H_2_O and one from surface oxygen plus dissociated H [28,33]. These OH groups could substantially influence the wetting behavior, thermal stability, and catalytic activity of metal oxides, and so forth, some of which arise from the hydrogen bonding between OH groups and molecules and OH-induced electronic structure changes of catalyst surfaces [33]. Therefore, investigation of the interaction of adsorbed H_2_O and/or OH groups with CO on metal surfaces can help in understanding water gas shift reactions [70]. However, the OH-assisted pathway should start with the co-adsorption of CO*, OH*, and H*. OH groups, as an active hydrogen species derived from H_2_ or H_2_O derivatives, have been reported to play an important role in FTS activity and selectivity as well as in CO_2_ hydrogenation [71]. The role of OH groups as surface hydride species may be universal and involved in several oxygenated compound conversion reactions [33].

**Figure 6:**
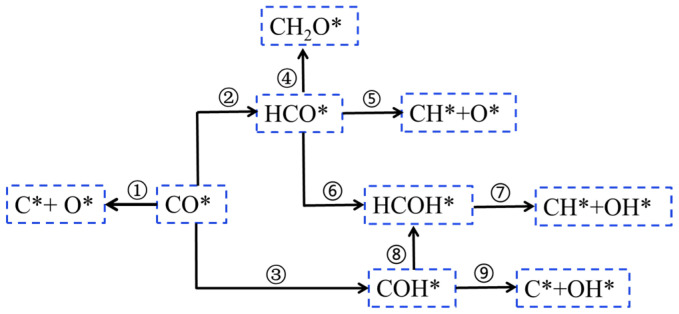
The elementary steps of OH-assisted CO-activated hydrogenation in the FTS reaction. Reproduced with permission from ref. [45]. Copyright (2010) Elsevier.

The presence of an OH group contributes to improve the C-O bond breaking ability compared to the co-adsorption with H* species, which leads to an increase in CH_X_ [33,61]. Therefore, in addition to unassisted CO activation and H-assisted CO activation pathway, Gunasooriya et al. [33] reported an OH-assisted CO pathway. As shown in Figure 6, CO* intermediate can be directly dissociated into C* and O* [33] (i.e., the carbide mechanism, step 1, Figure 6). Meanwhile, both hydrogenated CO* and RCO* preferentially hydrogenate at the C* species due to the geometry of the OH groups, forming the intermediate HCO* species [33] (step 2, Figure 6). The intermediates of HCO* and formaldehyde (H_2_CO*) intermediates are the co-adsorbed CO* and H* formation routes demonstrated by Mitchell et al. (step 4, Figure 6) [72]. The reaction forming CH* and O* from the HCO* intermediate dissociate directly (step 5, Figure 6), and CH* species continue to hydrogenate to form CH_2_*. HCO* species are hydrogenated to hydroxylmethylene (HCOH*), a reaction mechanism recently proposed by Gunasooriya et al. [33,73]. HCOH* species form CH* and OH* by double-assisted dissociation (step 7, Figure 6). Another alternative pathway is through hydrogenation of the oxygen end of CO* to form hydroxyl-carbene (COH*) as an intermediate (step 3, Figure 6), further dissociating into OH* and C* species [74] (step 9, Figure 6). This pathway avoids the formation of H_2_CO. CO* may be hydrogenated by the C* or O* species to form HCO* or COH* species as reaction intermediates, respectively. With respect to the H-assisted routes, the formations of HCO* and COH* are an endothermic process, and the formation of COH* is kinetically more favorable than the formation of CHO* species and the direct dissociation CO. In both cases, the formation of intermediates (CHO* and COH*) has a subsequent dissociation step leading to the formation of CH* or OH* species. In the FTS process, the OH* group on the surface is the effective hydrogenated substance.

### 2.5. O-Assisted CO Activation

H_2_O dissociates more readily on the surface of Fe- and Co-based catalysts with the O-assisted, and removing the oxygen atoms in an adsorbed state would be beneficial for refreshing the surface and preventing the surface from oxidating. Moreover, the breaking of the O-H bond is facilitated by O atoms and H_2_O molecules. In the CO activation reaction, it has recently been proposed that H_2_O plays a key role due to its ability to supply hydrogen to the O atoms in CO [65]. H_2_O and CO_2_ act as the main deoxygenation pathways in the FTS reaction, leading to the belief that most oxygenated products (CO activation) leave the catalyst surface in the form of H_2_O [75,76,77]. The use of H_2_O and CO_2_ as supports of O atoms in the unassisted CO activation and H-assisted CO activation steps is worthy of discussion. H-assisted CO dissociation removes O atoms as H_2_O, while direct dissociation forms chemisorbed oxygen atoms that desorb as CO_2_. Direct CO dissociation routes are minor contributors to monomer formation on Fe-based and may become favored at high temperatures on alkali-promoted catalysts, but not on Co-basted catalysts, which remove oxygen predominantly as H_2_O because of the preponderance of H-assisted CO dissociation routes [45]. Therefore, it is necessary to explore the potential mechanism for the removal of surface O* on CO-activated hydrogenation. Here, four kinds of O removal pathways under an FTS environment were roughly investigated. In the removing routes through H_2_O molecules, O-H bond formation was followed by two viable pathways: one is that the OH hydrogenates sequentially, the other is that the OH interacts with another OH [49]. The removal of CO_2_ form could occur in a direct way in which surface O atoms react with adsorbed CO, or in an indirect way with COOH* as an intermediate followed by dehydrogenation [33,49]. 

H_2_O molecules can dissociate directly into OH* and H* species (Equation (5)). OH* species react rapidly and disproportionately to produce H_2_O molecules (Equation (6)), which immediately dissociate into OH* and H* species [18,19]. Provided that Equations (5) and (6) react fast enough, O* and OH* species are considered to be quasi-equilibrium under the FTS reactions. It is worth noting that when O-H forms a bond, the reaction between an OH* intermediate and another OH* is superior to that with H, which is due to the latter needing higher barrier and endothermic energy. The OH* disproportionation reactions are kinetically unfavorable and thermodynamically more favorable. Therefore, it is the OH* rather than the O* species that is pre-existing on the catalyst surface, which is readily hydrogenated to form OH* (Equation (7)). Meanwhile, the conversion of O* to OH* is faster than the reaction of O* with CO* to form CO_2_ (Equation (8)) [34]. As in the pathways of H_2_O formation, a hanging chemisorbed H* and O* are produced by rapid reaction on the catalyst surface. One is that OH* species disproportionation react to form H_2_O molecules and O* species [17]; the other is that the OH* hydrogenates sequentially. This suggests that H_2_O is one of the main pathways for the removal of O* species. In addition, CO_2_ is also one of the important pathways for the removal of oxygen. The removal of surface oxygen in the form of CO_2_ was explored, in which the direct reaction of surface O* with CO* and indirect route through COOH* intermediate was included (COOH* dehydrogenation) [33]. In addition, COOH* and HCOOH* intermediates are formed through OH* and CO* species coupling and CO_2_ hydrogenation in two paths, in which the barrier and reaction energy are overcome differently (Equations (9) and (10)). As an alternative way to form CO_2_, the reaction between CO* and O* is the main pathway for the formation of CO_2_. Herein, we explored the removal of pre-adsorbed oxygen on Fe- and Co-based catalyst surfaces, and several pathways were investigated, including both direct and indirect routes for generating H_2_O and CO_2_. The removal mechanism shows diversity over different catalysts. The removal of O atoms is different on the surface of Fe- and Co-based catalysts, where O atoms are removed to form H_2_O and/or CO_2_ on Fe-based catalysts, while Co-based catalysts only generate H_2_O as O atom removal products [78]. Finally, the CO*-derived intermediates can also form CO_2_ via Boudouard reaction (CO* + CO* → C* + CO_2_*). These reaction pathways facilitate the exchange of chemisorbed O* species between CO_2_ and H_2_O. However, in actual reactions, the surface species are mobile and interact with each other, stabilizing reaction intermediates to different extents and thus likely influencing the preferred CO activation mechanisms [36,43]. Our work provides knowledge about the mechanisms of O removal and CO_2_ and H_2_O formation: the removal of adsorbed oxygen on the surface may be necessary for refreshing the catalyst surfaces and protecting the catalysts from further oxidation.

H_2_O ↔ OH* + H* (5)

2OH* ↔ H_2_O + O* (6)

O* + H* ↔ OH* + *(7)

CO* + O* ↔ CO_2_* + O* (8)

CO* + OH* ↔ COOH* + *(9)

CO_2_ + 2H* ↔ HCOOH* (10)

## 3. Concluding Remarks and Future Perspectives

The mechanism of the effect of H_2_O on the FTS performance of Fe- and Co-based catalyst surfaces remains unclear and requires further investigation. It is worth noting that in-depth understanding and accurate control of the intermediates formed in the reaction and the reaction path formation are required to significantly improve catalytic performance. In view of the current problems, this paper uses the intrinsic mechanism of the H_2_O molecule and derivatives as an accelerator on the surface of Fe- and Co-based catalysts to stimulate a better understanding of various H_2_O effects in various reaction systems. In this paper, the effects of H_2_O molecules and H_2_O derivatives (H*, OH* and O* species) on CO activation reactions and reaction intermediates are reviewed. We can conclude form this review article as follows:

- The positive mechanism of action of H_2_O facilitates partial transport of syngas and hydrocarbons and influences the kinetics of the reaction. H_2_O increased the coverage C* species by preferentially increasing the rate of CO activation.
- H-assisted CO dissociation activation is easier on the surface of Fe- and Co-based catalysts than unassisted CO. H-assisted pathways are more advantageous for kinetic formation of COH* species than direct dissociation of CO. The co-adsorption H* has a stabilizing effect in the system, and increasing the adsorption energy of CO helps its recombination.
- OH groups can induce changes in the electronic structure of catalyst surfaces. The presence of OH group contributes to improve the C-O bond breaking ability and can also be used as a surface hydrogenated species of active hydrogen species and participate in the conversion reaction of oxygen-containing compounds.
- The O atoms promote the breaking of O-H bond. H_2_O and CO_2_ molecules were discussed as carriers of O atoms. The removal pathways of four oxygen species in FTS were analyzed.

Since H_2_O may be present in FTS as H_2_O molecules, it may also dissociate into OH/OH^−^ and H/H^+^ on the catalyst surface, affecting the secondary reaction in FTS as well as the activity and selectivity of the product. We believe that studies on the H_2_O-derivatives need to help understand the mechanism of the effect of H_2_O on the activity and selectivity of FTS. This paper reviews CO activation on the surface of Fe- and Co-based catalysts from four aspects: H_2_O-assisted, H-assisted, OH-assisted, and O-assisted. Our group reported a series of work such as hydrophilic modification of catalyst surfaces, functional group modification, and preparation of catalysts with special morphology and achieved good experimental results [79,80,81,82,83]. Based on the experimental work of our group, combined with theoretical calculations, the influence of H_2_O-derivative (H_2_O molecules, H*, OH*, O*) intermediates on the formation and product distribution of FTS reaction intermediates was explored. By using different methods to prepare catalysts, surface modification, pre-treatment conditions, and reaction conditions, the formation and quantity of H_2_O molecule intermediates, H*, OH*, and O* species on the surface of Fe- and Co-based catalysts will be controlled, and the product distribution will be regulated. The current work explores the addition of reagents containing hydrophobic groups to the pretreatment solution for the preparation of Fe-based catalysts. The material preparation has been completed, and application and characterization data are being compiled. Finally, more research work has to be conducted to show clearly how the structure and surface properties of the supports could play a role in the way H_2_O affects the FTS performance of Fe- and Co-based catalysts.

## Data Availability

The data generated during the current study are available from the corresponding author upon reasonable request.
